# Gram-Negative Infections in Orthopedic Surgery: Current Challenges for Anesthesiologists and Intensive Care Physicians—A SANRA Narrative Review

**DOI:** 10.3390/jcm15218615

**Published:** 2026-11-05

**Authors:** Gabriele Melegari, Andrea Tognù, Eros Pilia, Domenico Santonastaso, Elisabetta Bertellini, Alberto Barbieri

**Affiliations:** 1 Department of Anesthesia and Intensive Care, ASST G.Pini-CTO, 20122 Milan, Italy; andrea.tognu@asst-pini-cto.it; 2 Department of Medical Science and Public Health, University of Cagliari, 09042 Cagliari, Italy; dotterospilia@gmail.com; 3 Department of Anesthesia and Intensive Care, Bufalini Hospital Azienda USL Cesena, 41125 Modena, Italy; domenicopietro.santonastaso@auslromagna.it; 4 School of Medicine, University of Modena and Reggio Emilia, 41121 Modena, Italy; elisabettab53@gmail.com; 5 School of Anesthesia and Intensive Care, University of Modena and Reggio Emilia, 41121 Modena, Italy; alberto.barbieri@unimore.it

**Keywords:** Gram-negative infections, orthopedic surgery, prosthetic joint infection, fracture-related infection, sepsis, anesthesiology, intensive care, antimicrobial stewardship

## Abstract

**Background**: Gram-negative bacterial infections represent an increasingly relevant challenge in orthopedic surgery, particularly in complex patients exposed to major trauma, revision procedures, prolonged hospitalization, previous antimicrobial therapy, and intensive care. Although Gram-positive organisms remain predominant in prosthetic joint infections (PJIs) and fracture-related infections (FRIs), the emergence of multidrug-resistant (MDR) Gram-negative pathogens has important implications for perioperative management, antimicrobial prophylaxis, and treatment. **Objective**: This narrative review examines the epidemiology, pathogenesis, antimicrobial resistance, and management of Gram-negative orthopedic infections, with particular emphasis on issues relevant to anesthesiologists and intensive care physicians. **Methods**: A structured narrative literature search was conducted in PubMed/MEDLINE, Scopus, and Web of Science from database inception to July 2026, according to the principles of the Scale for the Assessment of Narrative Review Articles (SANRA). Original studies, systematic reviews, meta-analyses, guidelines, consensus statements, and relevant narrative reviews addressing Gram-negative infections in orthopedic surgery were considered. **Results**: Gram-negative pathogens account for a minority of orthopedic infections but become increasingly relevant in complex PJIs, FRIs, open fractures, revision surgery, and patients with substantial healthcare exposure. *Enterobacterales*, *Pseudomonas aeruginosa*, and *Acinetobacter baumannii* are among the principal pathogens involved. Their clinical relevance is amplified by biofilm formation and increasingly complex antimicrobial resistance mechanisms. Perioperative antibiotic prophylaxis should remain individualized in patients colonized or previously infected with MDR Gram-negative organisms, avoiding indiscriminate broad-spectrum prophylaxis. Once infection is established, management requires adequate microbiological sampling, surgical source control, and susceptibility-guided antimicrobial therapy. New β-lactam/β-lactamase inhibitor combinations and cefiderocol have expanded therapeutic options against resistant Gram-negative pathogens; however, evidence specifically supporting cefiderocol for bone and joint infections remains limited. **Conclusions**: Gram-negative orthopedic infections represent an emerging challenge at the intersection of orthopedic surgery, infectious diseases, anesthesiology, and critical care. Early recognition of high-risk patients, individualized perioperative strategies, effective source control, resistance-guided antimicrobial therapy, and multidisciplinary management are essential.

## 1. Introduction

**Figure 1:**
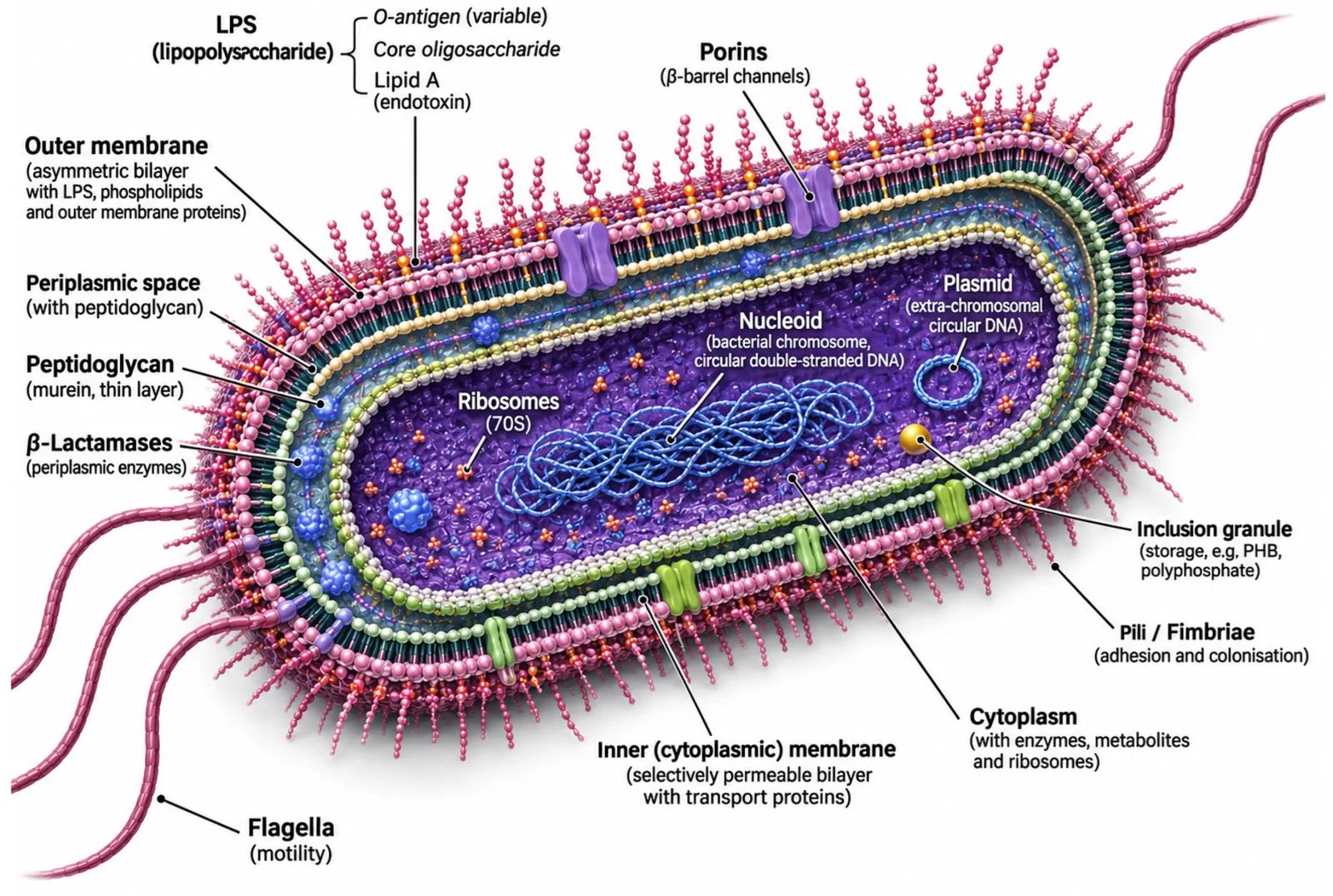
Structural organization and major biological features of Gram-negative bacteria. Gram-negative bacteria are characterized by a complex cell envelope consisting of an **inner cytoplasmic membrane**, a **periplasmic space containing a thin peptidoglycan layer**, and an **outer membrane**. The outer membrane contains **lipopolysaccharide (LPS)**, whose lipid A component is responsible for endotoxin activity and contributes to the host inflammatory response during severe infection. **Porins** regulate the passage of small hydrophilic molecules, including several antimicrobial agents, whereas alterations in porin expression or structure may contribute to antimicrobial resistance. The periplasmic space may contain **β-lactamases** and other enzymes capable of inactivating antibiotics. Surface structures such as **pili and fimbriae** facilitate adhesion to host tissues and abiotic surfaces and may contribute to colonization and biofilm formation, while **flagella**, when present, promote motility and bacterial dissemination. Together, these structural characteristics contribute to the pathogenicity, inflammatory potential, environmental adaptability, and antimicrobial resistance of Gram-negative bacteria.

Gram-negative bacteria represent a heterogeneous group of microorganisms characterized by a distinctive cell envelope architecture. Unlike Gram-positive bacteria, which possess a thick peptidoglycan layer, Gram-negative organisms have a relatively thin peptidoglycan layer located within the periplasmic space and an additional outer membrane. This outer membrane contains lipopolysaccharide (LPS), whose lipid A component plays a major role in the host inflammatory response to severe Gram-negative infections. The outer membrane also acts as a permeability barrier and, together with porins, efflux pumps and several enzymatic resistance mechanisms, contributes to the intrinsic or acquired reduced susceptibility of many Gram-negative organisms to antimicrobial agents (Figure 1) [1,2].

Gram-negative bacteria are responsible for a substantial proportion of healthcare-associated infections (HAIs), particularly among hospitalized and critically ill patients. *Enterobacterales*, including *Escherichia coli*, *Klebsiella pneumoniae*, *Enterobacter* spp., *Serratia* spp. and *Proteus* spp., together with non-fermenting Gram-negative bacilli such as *Pseudomonas aeruginosa* and *Acinetobacter baumannii*, are among the microorganisms most frequently encountered in the hospital environment [2,3,4]. Their relevance is particularly evident in urinary tract infections, hospital- and ventilator-associated pneumonia, bloodstream infections, intra-abdominal infections and infections associated with invasive devices. Prolonged hospitalization, intensive care unit (ICU) admission, previous antimicrobial exposure, invasive procedures and colonization with multidrug-resistant organisms further increase the probability of Gram-negative infection.

Orthopedic surgery has traditionally represented a somewhat different microbiological environment. Surgical-site infections (SSIs), fracture-related infections (FRIs) and prosthetic joint infections (PJIs) have historically been dominated by Gram-positive microorganisms [5]. *Staphylococcus aureus* and *coagulase-negative staphylococci* remain particularly important because of their ability to adhere to foreign materials and establish biofilm-associated infections on orthopedic implants. Consequently, both preventive strategies and empirical antimicrobial approaches in orthopedic surgery have traditionally focused heavily on Gram-positive pathogens.

Nevertheless, Gram-negative bacteria constitute a clinically relevant and potentially increasing component of the microbiological spectrum of orthopedic infections. *Enterobacterales* and *P. aeruginosa* are repeatedly identified in SSIs, PJIs and FRIs, either as single pathogens or as part of polymicrobial infections. Their relative contribution may be particularly relevant in selected populations, including patients with open fractures, traumatic injuries, repeated surgical procedures, prolonged hospitalization, previous antimicrobial exposure, chronic wounds or soft-tissue damage, and patients requiring intensive care. The epidemiological profile may therefore differ substantially between an uncomplicated primary elective arthroplasty and complex orthopedic patients exposed to repeated healthcare interventions [6].

More importantly, the global spread of antimicrobial resistance has profoundly changed the clinical significance of Gram-negative infections. Extended-spectrum β-lactamase-producing *Enterobacterales*, carbapenem-resistant *Enterobacterales*, multidrug-resistant *P. aeruginosa* and carbapenem-resistant *A. baumannii* represent major therapeutic challenges in contemporary hospital practice [7].

Understanding the microbiological and clinical characteristics of Gram-negative infections is therefore becoming an important component of modern perioperative and critical care medicine in orthopedic surgery.

Thus, although Gram-positive organisms remain central to the epidemiology of orthopedic infections, Gram-negative pathogens should no longer be considered a minor clinical concern in this setting. Their increasing relevance reflects the convergence of changing patient characteristics, greater surgical complexity, healthcare exposure, implant-associated infection and the global expansion of antimicrobial resistance [8]. These considerations are particularly important for anesthesiologists and intensive care physicians, who increasingly participate in the perioperative management of high-risk orthopedic patients and may be directly involved in the treatment of severe postoperative infections, sepsis and septic shock. This narrative review examines the emerging role of Gram-negative pathogens in orthopedic infections, focusing on their epidemiology, antimicrobial resistance patterns and clinical implications, with particular attention to the challenges relevant to anesthesiologists and intensive care physicians.

## 2. Materials and Methods

### 2.1. Literature Search Strategy

This narrative review was conducted based on the principles outlined by the Scale for the Assessment of Narrative Review Articles (SANRA) [9]. We conducted a structured literature search to identify relevant publications on Gram-negative infections in orthopedic surgery and their implications for perioperative and critical care medicine. A narrative approach was selected instead of a systematic one because the topic encompasses diverse evidence from microbiology, orthopedic surgery, infectious diseases, perioperative medicine, and intensive care, making quantitative synthesis impractical and potentially incomplete. Electronic searches were conducted in PubMed/MEDLINE, Scopus, and Web of Science, from the inception of these databases until July 2026. Additional relevant publications were identified through manual screening of reference lists from eligible articles and major international guidelines. The search strategy employed a combination of controlled vocabulary terms and free-text keywords related to Gram-negative infections, orthopedic surgery, perioperative medicine, and intensive care. The search terms included: “Gram-negative bacteria,” “Gram-negative infection,” “orthopedic surgery,” “prosthetic joint infection,” “fracture-related infection,” “surgical site infection,” “multidrug-resistant organisms,” “*Enterobacterales*,” “*Escherichia coli*,” “*Klebsiella pneumoniae*,” “*Pseudomonas aeruginosa*,” “*Acinetobacter baumannii*,” “anesthesiology,” “perioperative care,” “critical care,” “intensive care,” “sepsis,” and “antimicrobial resistance.” To provide an overview of the available literature, an exploratory PubMed search combining terms related to prosthetic joint infections and Gram-positive and Gram-negative bacteria yielded 64,317 records over the preceding ten years. This preliminary search was intended to illustrate the breadth of the published literature rather than to establish a formal systematic screening process. Given the narrative nature of the review and the heterogeneity of the available evidence, we selected publications based on clinical relevance, methodological quality, and contribution to the manuscript’s objectives, with particular emphasis on Gram-negative pathogens, antimicrobial resistance, and perioperative and intensive care management. Accordingly, no formal PRISMA-based selection process was undertaken, consistent with the narrative approach adopted in accordance with SANRA principles.

### 2.2. Eligibility Criteria

We considered original research articles, systematic reviews, meta-analyses, narrative reviews, consensus statements, clinical practice guidelines, and position papers published in English as eligible for inclusion. Special emphasis was placed on studies that explored the epidemiology, microbiology, antimicrobial resistance, diagnosis, prevention, perioperative management, and critical care implications of Gram-negative infections in orthopedic patients. We excluded studies that focused solely on Gram-positive pathogens without relevant discussions of Gram-negative microorganisms, as well as non-human studies, conference abstracts lacking full-text availability, and publications that did not provide sufficient methodological detail.

### 2.3. Study Selection and Data Synthesis

The literature obtained was screened for relevance by evaluating the title, abstract, and full text. Due to the narrative nature of this review, we did not conduct a formal quantitative synthesis or meta-analysis. Instead, the evidence was critically assessed and synthesized narratively to provide a comprehensive overview of current knowledge. We prioritized high-quality evidence, including international guidelines, multicenter observational studies, systematic reviews, meta-analyses, and landmark investigations related to orthopedic infections, antimicrobial resistance, and the management of critically ill surgical patients. When available, we included contemporary data from major surveillance systems and international organizations, such as the European Centre for Disease Prevention and Control (ECDC), the World Health Organization (WHO), and relevant orthopedic and infectious disease societies.

## 3. Results

### 3.1. Pathogenesis of Orthopedic Infection: Gram-Positive Versus Gram-Negative Bacteria

Orthopedic infections arise from a complex interplay of factors, including bacterial virulence, host susceptibility, local tissue conditions, and the presence of foreign materials. Microorganisms can reach the surgical site through direct contamination during surgery or trauma, by spreading from nearby tissues, or, less frequently, through hematogenous dissemination from a distant infection site. The presence of prosthetic materials or fixation devices influences these interactions by providing a surface for bacterial adhesion and subsequent biofilm development [10].

Gram-positive bacteria, particularly *Staphylococcus aureus* and *coagulase-negative staphylococci*, have historically been the primary pathogens associated with orthopedic implant infections. Their ability to adhere to host extracellular matrix proteins and implant surfaces through specific adhesins plays a significant role in their pathogenicity. After attachment, bacterial aggregation and biofilm maturation enhance persistence on prosthetic materials, reduce vulnerability to host immune responses and antimicrobial treatments, and contribute to the development of chronic or recurrent infections. This biological behavior underscores the notable role of staphylococci in prosthetic joint infections and fracture-related infections [11].

While Gram-negative bacteria share some pathogenic mechanisms with Gram-positive bacteria, they present additional distinct features. Their outer membrane contains lipopolysaccharide (LPS), which interacts with innate immune receptors, particularly Toll-like receptor 4, promoting the release of pro-inflammatory mediators. In severe infections, this response can lead to endothelial dysfunction, vasodilation, altered microcirculation, and organ dysfunction. Several Gram-negative pathogens relevant to orthopedic infections, including *Pseudomonas aeruginosa*, *Klebsiella pneumoniae*, and members of the *Enterobacterales*, possess adhesins, pili, secretion systems, and other virulence factors that facilitate tissue colonization and persistence [12].

Importantly, biofilm formation is not exclusive to Gram-positive organisms; Gram-negative bacteria can also establish mature biofilms on orthopedic implants and devitalized tissues. *P. aeruginosa*, in particular, can produce a highly structured extracellular matrix and develop phenotypically diverse bacterial populations with reduced susceptibility to antibiotics. In infections associated with Gram-negative implants, biofilm-mediated persistence can coexist with intrinsic and acquired antimicrobial resistance mechanisms, including reduced outer-membrane permeability, efflux pumps, and β-lactamase production [13].

**Figure 2:**
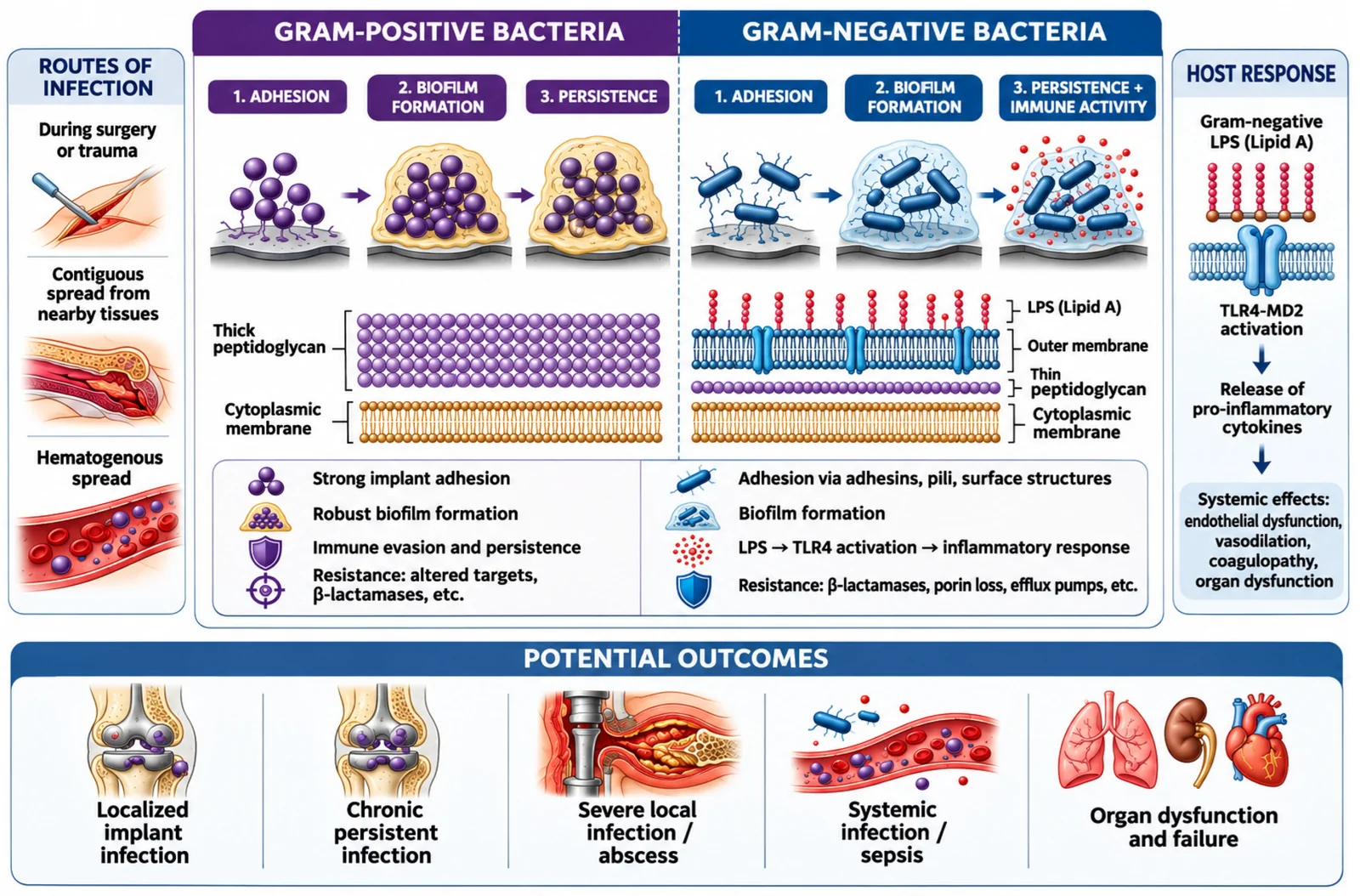
Pathogenesis of Gram-positive and Gram-negative orthopedic infections. Orthopedic infections may arise from direct contamination, contiguous spread, or hematogenous dissemination. Both Gram-positive and Gram-negative bacteria can adhere to implant surfaces and form biofilms, promoting persistence and chronic infection. Gram-negative pathogens additionally exhibit distinctive features related to their outer membrane, LPS-mediated inflammatory response, and multiple antimicrobial resistance mechanisms. Depending on pathogen, host, and local factors, infection may remain localized or progress to sepsis and organ dysfunction.

Gram-positive infections are typically linked to strong implant adhesion and persistent biofilm-associated disease, Gram-negative infections combine the ability to adhere and form biofilms with a complex outer membrane that activates the innate immune response and increasingly relevant mechanisms of antimicrobial resistance (Table 1). These characteristics can influence clinical presentation, choices of antimicrobial therapy, strategies for source control, and, in severe cases, the risk of progression from localized orthopedic infections to systemic infections and organ dysfunction (Figure 2).

### 3.2. Prosthetic Joint and Fracture-Related Infections: Definitions and Epidemiology

Prosthetic joint infection (PJI) and fracture-related infection (FRI) are two major types of implant-associated infections in orthopedic surgery. While they share several pathogenic mechanisms, particularly bacterial adhesion and biofilm formation on foreign materials, they arise in different clinical contexts and are defined by specific diagnostic criteria.

Prosthetic joint infection occurs in the joints with implants and their surrounding tissues. Current diagnostic definitions integrate major criteria such as a sinus tract that communicates with the prosthesis or microbiological confirmation from multiple samples taken near the prosthesis, along with clinical, laboratory, synovial, microbiological, and histological findings. PJIs can occur due to perioperative contamination, contiguous spread, or hematogenous seeding of an implanted prosthesis. Based on the timing and method of infection, PJIs can present as early postoperative, delayed/chronic, or acute hematogenous infections [14].

Despite advancements in surgical techniques, antimicrobial prophylaxis, and infection prevention strategies, PJI remains one of the most serious complications of joint arthroplasty. Its incidence is generally estimated to be around 1–2% following primary hip or knee arthroplasty, with higher rates observed after revision procedures and in patients with multiple comorbidities or prior surgeries. Given the steadily increasing number of joint replacements performed worldwide, even a relatively low incidence results in a significant and growing clinical and healthcare burden [15].

Fracture-related infection is defined as an infection that occurs in the presence of a fracture, regardless of whether internal fixation is used. International consensus criteria differentiate confirmatory signs, such as a fistula, sinus, or wound breakdown communicating with the fracture or implant, purulent drainage, and microbiological or histological evidence of infection, from suggestive clinical, radiological, and laboratory findings that require further investigation. In contrast to PJI, FRI often occurs following traumatic events, where factors such as contamination, soft-tissue damage, impaired vascularization, fracture instability, and repeated surgical procedures can contribute to the development of infection [16,17].

The incidence of FRI varies significantly based on the characteristics and severity of the fracture. Infection rates after closed fractures treated with internal fixation are generally low, around 1–2%. However, this risk increases substantially for open fractures and cases involving severe soft-tissue injury, reaching 20–30% or more in the most extreme situations. Factors such as open fractures, high-energy trauma, extensive tissue damage, vascular compromise, prolonged hospitalization, repeated surgeries, and intensive care exposure can also alter the microbiological profile and increase the likelihood of Gram-negative and multidrug-resistant pathogens [18,19]. Thus, PJI and FRI are distinct but overlapping clinical entities. While Gram-positive organisms, particularly *Staphylococcus aureus* and *coagulase-negative staphylococci*, remain the most common, Gram-negative pathogens also represent a significant aspect of both infections especially in complex patients with considerable healthcare exposure, traumatic injuries, multiple procedures, or previous antimicrobial therapy (Table 2).

### 3.3. Microbiological Spectrum of PJI and FRI: Where Gram-Negative Pathogens Emerge

The range of microorganisms involved in orthopedic infections varies with several factors, including the type of surgery performed, the route of infection, the patient’s characteristics, prior healthcare experiences, and the extent of local tissue damage. While Gram-positive organisms, especially *Staphylococcus aureus* and *coagulase-negative staphylococci*, are the most common culprits, Gram-negative bacteria also play a significant role in both PJIs and FRIs.

In prosthetic joint surgery, Gram-negative bacteria account for approximately 10% to 20% of prosthetic joint infections (PJIs), although variability exists across patient cohorts [20,21]. *Enterobacterales*, particularly *Escherichia coli*, *Klebsiella pneumoniae*, *Enterobacter* spp., and *Proteus* spp., together with *Pseudomonas aeruginosa*, represent the most frequently identified pathogens. Recent European multicenter data showed that Gram-negative organisms accounted for approximately 9% of culture-confirmed hip and knee PJIs, with *E. coli* and *P. aeruginosa* among the predominant microorganisms. Gram-negative PJIs appear particularly relevant after hip arthroplasty, revision procedures, and in elderly patients with multiple comorbidities [15]. Diabetes, obesity, previous antimicrobial exposure, recurrent hospitalization, urinary tract infection or colonization, and repeated surgical procedures may further increase the likelihood of Gram-negative involvement.

The microbiological spectrum of fracture-related infections is generally broader. Although *S. aureus* remains the leading pathogen in most series, Gram-negative bacteria may account for a substantial proportion of isolates, particularly those from the *Enterobacterales* and *P. aeruginosa*. In a major trauma-center cohort, Gram-negative organisms accounted for 39.7% of recovered microorganisms, with *P. aeruginosa*, *Enterobacter* spp., and *E. coli* the most frequently identified Gram-negative pathogens [22,23].

The phenotype associated with Gram-negative FRI appears particularly related to the complexity of trauma and its surgical management. Patients with multisystem trauma, open fractures requiring soft-tissue reconstruction, temporary external fixation followed by definitive internal fixation, repeated debridement, skin grafting or flap coverage, and prolonged hospitalization show greater representation of Gram-negative organisms. Recent data from a Level I trauma center showed Gram-negative microorganisms in approximately one-quarter of FRI patients; multisystem trauma, external fixation, skin grafting and flap coverage were significantly more frequent among patients with Gram-negative infection. Previous studies similarly identified initial external fixation, requirement for soft-tissue coverage, and hospitalization longer than 10 days as factors associated with Gram-negative FRI [1].

Therefore, Gram-negative pathogens do not replace the traditional Gram-positive microbiology of orthopedic infections but emerge within specific patient–procedure profiles [5]. Their relevance progressively increases from uncomplicated elective arthroplasty toward revision surgery, complex fracture fixation and major orthopedic trauma, particularly when repeated surgery, extensive soft-tissue reconstruction and prolonged healthcare exposure coexist. Recognizing these clinical phenotypes may help anesthesiologists and intensive care physicians identify patients who may need broader microbiological assessment and closer perioperative infectious surveillance.

### 3.4. Host Response to Gram-Positive and Gram-Negative Orthopedic Infections

The clinical consequences of orthopedic infection depend not only on bacterial virulence and local tissue involvement but also on the magnitude and regulation of the host immune response. Although Gram-positive and Gram-negative bacteria activate partially distinct innate immune pathways, both may ultimately induce systemic inflammation, endothelial dysfunction, microcirculatory impairment, coagulation abnormalities, and organ dysfunction when infection extends beyond the local compartment.

In Gram-positive infections, pathogen-associated molecular patterns such as peptidoglycan and lipoteichoic acid are recognized predominantly through Toll-like receptor 2 (TLR2)-dependent pathways. *Staphylococcus aureus* may also release toxins and other virulence factors that amplify immune activation and tissue damage. Activation of monocytes, macrophages, and neutrophils promotes the release of pro-inflammatory mediators, including tumor necrosis factor-α (TNF-α), interleukin-1β (IL-1β), and IL-6. Therefore, despite their frequent association with localized and chronic implant-associated infections, Gram-positive organisms can also produce severe systemic disease, bacteremia, sepsis and septic shock [24,25].

In Gram-negative infections, lipopolysaccharide (LPS) represents a major trigger of the innate immune response. The lipid A component of LPS is recognized through the LPS-binding protein/CD14/TLR4 complex, activating intracellular signaling pathways and promoting cytokine release. When bacterial burden and host response become excessive, this process may contribute to vasodilation, increased endothelial permeability, altered microvascular perfusion, and activation of coagulation, providing a mechanistic link between localized infection, systemic inflammation, and multiple-organ dysfunction [26].

Clinical evidence suggests that these biological differences may translate into different inflammatory profiles. A recent systematic review and meta-analysis found higher circulating concentrations of procalcitonin, C-reactive protein, and TNF-α in Gram-negative sepsis than in Gram-positive sepsis. Gram-negative infection was also associated with a higher occurrence of severe sepsis or septic shock. However, no significant difference in survival was observed between Gram-negative and Gram-positive sepsis, emphasizing that Gram classification alone does not determine patient prognosis [27].

For the anesthesiologist and intensive care physician, this distinction is clinically relevant but should not replace assessment of infection severity. Progression from an orthopedic infection to systemic disease is determined by the interaction between pathogen, bacterial burden, adequacy of source control, timing of antimicrobial therapy, and host vulnerability. Both Gram-positive and Gram-negative orthopedic infections may therefore progress from localized disease to bacteremia, sepsis, and organ dysfunction, requiring prompt recognition and multidisciplinary management.

### 3.5. Multidrug Resistance: An Emerging Challenge in Gram-Negative Orthopedic Infections

Antimicrobial resistance is a significant challenge in treating Gram-negative infections. The unique structure of the Gram-negative cell envelope creates an intrinsic permeability barrier. Additionally, acquired resistance can emerge from various mechanisms, often occurring together. These include the production of β-lactamases, loss or modification of porins, increased activity of efflux pumps, alterations in target sites, and the acquisition of mobile genetic elements that carry resistance genes. The combination of these mechanisms can lead to the development of multidrug-resistant (MDR), extensively drug-resistant (XDR), or difficult-to-treat resistant strains [28,29].

Among *Enterobacterales*, extended-spectrum β-lactamases (ESBLs) confer resistance to most penicillins and cephalosporins, while carbapenemase production may substantially compromise the activity of carbapenems and other β-lactams. Clinically relevant carbapenemases include *Klebsiella pneumoniae* carbapenemase (KPC), New Delhi metallo-β-lactamase (NDM), Verona integron-encoded metallo-β-lactamase (VIM), imipenemase (IMP), and oxacillinase-48-like (OXA-48-like) enzymes. In *Pseudomonas aeruginosa*, antimicrobial resistance is frequently multifactorial and may involve reduced outer-membrane permeability, particularly through loss or modification of the OprD porin, overexpression of multidrug efflux pumps, increased production of the chromosomal AmpC β-lactamase, and acquisition of carbapenemases. *Acinetobacter baumannii* can similarly accumulate multiple intrinsic and acquired resistance mechanisms, with carbapenem-resistant *A. baumannii* (CRAB) representing one of the most challenging antimicrobial-resistant phenotypes encountered in healthcare-associated infections [30,31].

The significance of these mechanisms is closely related to orthopedic infections. Periprosthetic joint infections (PJI) and fracture-related infections (FRI) often require extended antimicrobial treatment due to the presence of biofilm, poorly vascularized tissues, foreign materials, and the need for repeated surgical procedures. In this context, antimicrobial resistance decreases the number of available agents that can effectively combat infections while providing adequate penetration in bone and tissue and ensuring tolerability during prolonged treatment. Factors such as previous antibiotic use, repeated hospitalizations, revision surgeries, admissions to intensive care, and multiple surgical interventions can further select for or promote the acquisition of resistant microorganisms [32].

The magnitude of this problem varies substantially across geographical settings and patient populations. Nevertheless, MDR Gram-negative organisms are increasingly recognized in bone and joint infections, and resistant pathogens may negatively affect the probability of successful treatment. Their clinical relevance is reflected in the 2024 WHO Bacterial Priority Pathogens List, which classifies carbapenem-resistant *Acinetobacter baumannii*, third-generation cephalosporin-resistant *Enterobacterales*, and carbapenem-resistant *Enterobacterales* as critical-priority pathogens, while carbapenem-resistant *P. aeruginosa* remains a high-priority pathogen [33,34].

In orthopedic patients, therefore, antimicrobial resistance represents more than a microbiological finding. It interacts with biofilm formation, surgical source control, previous antimicrobial exposure, and patient vulnerability, potentially resulting in prolonged hospitalization, repeated surgical interventions, limited therapeutic options, and increased treatment complexity. Early microbiological identification and susceptibility testing are consequently essential to guide targeted therapy and avoid unnecessary exposure to broad-spectrum antimicrobial agents [30].

### 3.6. From Targeted Perioperative Prophylaxis to Treatment of Multidrug-Resistant Gram-Negative Infections

The rising incidence of antimicrobial resistance poses serious challenges to standard antibiotic prophylaxis before surgery. When planning surgical procedures for high-risk patients, it is essential to consider factors such as prior microbiological isolates, recent antimicrobial use, healthcare exposures, and colonization with multidrug-resistant organisms [35].

Current European guidelines recommend targeted perioperative antibiotic prophylaxis for patients colonized with multidrug-resistant Gram-negative bacteria. However, evidence supporting this approach remains limited and varies by surgery type and specific resistance phenotype [29].

As a result, it is not advisable to routinely expand surgical prophylaxis solely based on the presence of multidrug-resistant Gram-negative colonization. Instead, tailored strategies should consider prior microbiological data, local epidemiology, the type and urgency of the surgery, and antimicrobial stewardship principles [36].

Once an orthopedic infection is established, combine antimicrobial therapy with appropriate surgical intervention to control the infection source. Whenever possible, samples from deep tissues or associated implants should be collected before starting antimicrobial treatment, especially in patients who are clinically stable. Afterward, tailor definitive therapy based on the identified pathogen, its antimicrobial susceptibility and resistance mechanisms, the site of infection, the chosen surgical strategy, and individual patient factors related to pharmacokinetics and pharmacodynamics [37]. This tailored approach is particularly crucial for prosthetic joint infections and fractures, where biofilm formation, foreign material, impaired tissue penetration, and the need for extended antimicrobial therapy can significantly reduce the likelihood of successful eradication.

The emergence of multidrug-resistant Gram-negative pathogens has substantially expanded the role of newer β-lactam/β-lactamase inhibitor combinations and other novel agents. Ceftazidime-avibactam, meropenem-vaborbactam, and imipenem-cilastatin-relebactam offer important therapeutic options against selected carbapenem-resistant *Enterobacterales*, particularly depending on the underlying carbapenemase, while ceftolozane-tazobactam remains a major option against difficult-to-treat resistant *Pseudomonas aeruginosa* [38]. The choice of therapy should therefore increasingly be guided not only by conventional susceptibility testing but also, when available, by characterization of the underlying resistance mechanism [39,40].

Severe orthopedic infections, particularly those associated with multidrug-resistant Gram-negative pathogens, may progress from localized infection to sepsis and septic shock, especially in patients undergoing complex revision surgery, repeated debridement, or prolonged hospitalization. For anesthesiologists and intensive care physicians, early identification of clinical deterioration is essential to prevent progressive organ dysfunction.

Perioperative assessments systematically evaluate hemodynamic stability, mental status, respiratory function, peripheral perfusion, and urine output. Early warning scores, such as the National Early Warning Score 2 (NEWS2), may help identify clinical deterioration but should not replace clinical judgment or a comprehensive assessment of organ dysfunction. Serial serum lactate measurements may provide additional information regarding tissue hypoperfusion and response to resuscitation, although lactate concentrations should always be interpreted within the overall clinical context. Inflammatory biomarkers, including C-reactive protein and procalcitonin, may complement clinical assessment but should not be used as isolated diagnostic criteria for sepsis [41].

In patients with suspected sepsis or septic shock, prompt microbiological sampling, timely administration of appropriate antimicrobial therapy, and effective surgical source control are fundamental. Obtain blood cultures before antimicrobial administration whenever feasible, provided sampling does not substantially delay treatment. In patients with septic shock or a high likelihood of sepsis, initiate antimicrobial treatment promptly, then optimize based on microbiological findings and susceptibility testing [42].

Hemodynamic management should follow established principles of sepsis resuscitation. Balanced crystalloids are generally preferred for initial fluid administration, while further fluid therapy should be guided by repeated clinical assessment and dynamic indices of fluid responsiveness. Norepinephrine is the first-line vasopressor in septic shock, with an initial mean arterial pressure target of approximately 65 mmHg, then individualized based on patient characteristics and organ perfusion. In patients with persistent hemodynamic instability, invasive arterial pressure monitoring and bedside echocardiography may help characterize circulatory dysfunction, assess ventricular performance, and guide fluid and vasoactive treatment [43,44].

From an anesthesiological perspective, patients undergoing surgical debridement or prosthetic revision in the presence of systemic infection require careful preoperative risk assessment and individualized intraoperative management. Anesthetic induction may precipitate significant hypotension in patients with sepsis-associated vasodilation or myocardial dysfunction. Consequently, adequate vascular access, continuous hemodynamic monitoring, and early availability of vasopressor support are particularly important. Coordinate antimicrobial administration timing and intraoperative microbiological sampling with the surgical and infectious disease teams, while avoiding unnecessary delays in treating unstable patients.

Postoperative intensive care management should focus on the early detection and treatment of organ dysfunction, including respiratory failure, acute kidney injury, and persistent circulatory instability. In critically ill patients, antimicrobial pharmacokinetics may change substantially due to altered volume of distribution, renal clearance, and tissue perfusion. Optimization of antibiotic exposure, including appropriate loading doses, prolonged or continuous β-lactam infusions when indicated, renal dose adjustment, and therapeutic drug monitoring where available, may therefore be particularly relevant.

Ultimately, successful management requires integration of hemodynamic stabilization, timely antimicrobial treatment, and definitive surgical source control. Multidisciplinary collaboration between anesthesiologists, intensivists, orthopedic surgeons, microbiologists, and infectious disease specialists remains essential, particularly in patients with complex implant-associated infections caused by multidrug-resistant Gram-negative organisms [45].

Cefiderocol is an innovative therapeutic option among recently introduced agents. It is a siderophore cephalosporin that uses bacterial iron-uptake systems to penetrate Gram-negative outer membranes. Once inside the periplasmic space, cefiderocol primarily binds to penicillin-binding protein 3, inhibiting bacterial cell-wall synthesis. This unique mechanism, along with its resistance to several clinically relevant β-lactamases, provides broad in vitro activity against challenging Gram-negative pathogens [46]. These include carbapenem-resistant *Enterobacterales*, multidrug-resistant *Pseudomonas aeruginosa*, carbapenem-resistant *Acinetobacter baumannii*, and *Stenotrophomonas maltophilia* [47,48]. Cefiderocol may be particularly beneficial when resistance mechanisms greatly limit conventional treatment options. However, it should not be viewed as a universal first-line therapy for multidrug-resistant Gram-negative infections. Current guidelines specify its use based on the pathogen type and resistance profile. For treatment of difficult-to-treat, resistant *Pseudomonas aeruginosa*, cefiderocol is a viable option if susceptibility is confirmed. For carbapenem-resistant *Acinetobacter baumannii*, use is typically reserved for infections that do not respond to other treatments or when resistance or intolerance limits alternative therapies. It is advisable to use cefiderocol as part of combination therapy in these situations. Therefore, susceptibility testing is crucial, especially because heteroresistance and resistance development during treatment have been reported [49].

Evidence on cefiderocol for bone and joint infections is considerably more limited than evidence for bloodstream, respiratory, and urinary tract infections. Most published data are case reports and small observational studies of osteomyelitis, prosthetic joint infections, or other complex implant-associated infections caused by extensively resistant Gram-negative organisms. These reports indicate that cefiderocol can achieve clinical and microbiological success in select patients, particularly for infections caused by carbapenem-resistant *A. baumannii* and difficult-to-treat *P. aeruginosa* [50]. However, this evidence is insufficient to justify its routine use in orthopedic infections. Consider its potential role as part of a multidisciplinary approach that includes appropriate surgical source control, susceptibility-guided antimicrobial therapy, optimized drug exposure, and effective antimicrobial stewardship. International guidelines and consensus documents provide complementary recommendations for the diagnosis, prevention, and treatment of orthopedic infections. Although most recommendations are not specifically focused on Gram-negative pathogens, they offer a framework for microbiological assessment, surgical source control, antimicrobial selection, and perioperative management. The principal recommendations and their practical implications are summarized in Table 3 [14,29,51,52] (Table 3).

## 4. Discussion

Gram-negative infections represent a clinically relevant, although still minority, component of the microbiological spectrum of orthopedic infections. The available evidence indicates that their importance becomes progressively greater in selected clinical scenarios characterized by complex trauma, revision surgery, repeated procedures, prolonged healthcare exposure, previous antimicrobial treatment, and extensive soft-tissue damage. This is particularly evident in fracture-related infections and in complex prosthetic joint infections, where the microbiological spectrum is broader, and the probability of polymicrobial or resistant infection increases [53].

A central finding of this review is that the clinical relevance of Gram-negative infection cannot be understood solely in terms of pathogen prevalence. Gram-negative bacteria combine the capacity for implant adhesion and biofilm formation with distinctive structural and resistance mechanisms, including outer-membrane permeability barriers, efflux systems, and β-lactamase production [54]. These characteristics may contribute to the persistence of implant-associated infections while also limiting available therapeutic options. The rise in multidrug-resistant and extensively drug-resistant strains worsens this issue, especially when biofilm formation, impaired tissue vascularization, retained foreign materials, and prolonged antimicrobial exposure coexist.

From a perioperative and critical care perspective, another key consideration is how pathogen biology interacts with the host response. Although Gram-positive and Gram-negative organisms activate partially different innate immune pathways, both can ultimately lead to systemic inflammation and organ dysfunction. Gram-negative infections may elicit a particularly prominent endotoxin-mediated inflammatory response via lipopolysaccharide signaling, but current evidence does not support a straightforward relationship between Gram classification and mortality. Therefore, pathogen type should complement, rather than replace, clinical assessment of infection severity, source control, organ dysfunction, and host vulnerability [55].

These observations have practical implications for perioperative management. Standardized antibiotic prophylaxis remains appropriate for most orthopedic procedures, but a purely uniform approach may be insufficient for selected patients with known colonization or prior infection with multidrug-resistant Gram-negative organisms [56]. Current European guidance supports the concept of targeted prophylaxis in selected colonized patients, although the evidence remains limited and strongly dependent on surgical setting and resistance phenotype [28,29]. Consequently, indiscriminate broadening of perioperative prophylaxis should be avoided, and decisions should integrate previous microbiology, local epidemiology, surgical complexity, and antimicrobial stewardship.

Once an infection is established, a multidisciplinary approach to treatment is essential. Adequate microbiological sampling and surgical intervention to control the source of the infection are crucial. Definitive antimicrobial therapy should be guided by susceptibility testing, resistance mechanisms, the site of infection, and pharmacokinetic/pharmacodynamic considerations [57]. New combinations of β-lactams and β-lactamase inhibitors, as well as siderophore cephalosporins, have expanded the options for treating resistant Gram-negative pathogens. Cefiderocol is particularly appealing due to its unique iron-transport-mediated entry into Gram-negative bacteria and its broad in vitro activity against carbapenem-resistant *Enterobacterales*, difficult-to-treat *Pseudomonas aeruginosa*, carbapenem-resistant *Acinetobacter baumannii*, and *Stenotrophomonas maltophilia* [58].

However, evidence regarding cefiderocol’s effectiveness in treating bone and joint infections is largely limited to case reports and small observational studies. Therefore, its use should be individualized and guided by susceptibility testing [59].

Taken together, these findings suggest that Gram-negative orthopedic infections should not be considered a separate microbiological niche, but rather a manifestation of increasing patient complexity, healthcare exposure, antimicrobial pressure, and surgical burden. For anesthesiologists and intensive care physicians, their relevance lies particularly in early risk recognition, perioperative antimicrobial decision-making, management of sepsis and organ dysfunction, and coordination with orthopedic surgeons, microbiologists, and infectious disease specialists.

Although newer β-lactam/β-lactamase inhibitor combinations and cefiderocol have expanded therapeutic options against multidrug-resistant Gram-negative pathogens, the evidence supporting their use specifically in bone and joint infections remains limited. Most available data derive from case reports, small retrospective case series, and heterogeneous observational studies, frequently involving patients with complex infections, previous antimicrobial failures, and multiple surgical interventions. The absence of randomized controlled trials specifically addressing prosthetic joint infections and fracture-related infections limits the possibility of drawing definitive conclusions regarding comparative efficacy, optimal treatment duration, and long-term clinical outcomes [60].

For cefiderocol, published reports suggest potential clinical and microbiological efficacy against difficult-to-treat pathogens, particularly carbapenem-resistant *Acinetobacter baumannii* and multidrug-resistant *Pseudomonas aeruginosa*. However, these findings should be interpreted cautiously because of small sample sizes, selection and publication biases, variable definitions of treatment success, and limited long-term follow-up. Furthermore, the frequent use of concomitant antimicrobial agents and surgical source control makes it difficult to determine the independent contribution of cefiderocol to favorable outcomes [61]. In a bicentric cohort of 15 patients with bone and joint infections, Rempenault et al. reported clinical cure in 7 of 9 patients treated with ceftazidime-avibactam and 3 of 5 patients treated with ceftolozane-tazobactam, while one additional patient received both agents sequentially [62]. Although these findings suggest potential therapeutic utility, the small sample size and absence of a comparator group limit their generalizability. Additional uncertainties concern bone penetration, prolonged treatment safety, and the potential emergence of resistance during therapy. More recently, a French multicenter retrospective study evaluated cefiderocol in 45 patients with bone and joint infections, including 20 implant-associated infections [63]. Surgical management was performed in 82% of patients, and cefiderocol was administered in combination with other antimicrobial agents in 89%. Treatment failure was reported in 49% of patients, while 22% died during follow-up. These findings highlight the complexity of infections treated with cefiderocol and contrast with the favorable outcomes described in earlier individual case reports. However, the observational design, heterogeneous infection types, frequent combination therapy, and concomitant surgical interventions prevent definitive conclusions regarding the independent efficacy of cefiderocol.

Importantly, randomized controlled trials specifically evaluating these agents in prosthetic joint infections or fracture-related infections remain unavailable in the evidence identified. Therefore, although emerging antimicrobial agents provide additional treatment options for selected multidrug-resistant Gram-negative infections, their use in orthopedic infections should be guided by susceptibility testing, resistance mechanisms, surgical source control, and multidisciplinary clinical assessment rather than extrapolation from trials conducted in other infectious syndromes.

Similar limitations apply to newer β-lactam/β-lactamase inhibitor combinations, for which evidence in orthopedic infections is largely extrapolated from studies conducted in other infectious syndromes. Consequently, direct clinical evidence supporting the use of these agents in bone and joint infections remains limited and predominantly observational. Their use should therefore be individualized according to microbiological susceptibility, resistance mechanisms, patient characteristics, and surgical management, within a multidisciplinary approach involving orthopedic surgeons, infectious disease specialists, and intensive care physicians.

### Limitations

This review has several limitations. First, its narrative design does not allow for formal quantitative synthesis, pooled effect estimates, or the assessment of publication bias. The literature on Gram-negative orthopedic infections is highly heterogeneous, with varying definitions, surgical populations, geographical settings, resistance patterns, and microbiological methods, which limits direct comparisons between studies.

Second, much of the available evidence comes from retrospective cohorts, single-center studies, and selected populations, such as major trauma or referral centers, where the prevalence of Gram-negative and multidrug-resistant organisms may be higher than in routine elective orthopedic practice. There is limited evidence specifically addressing anesthesiology and intensive care management, making it necessary to extrapolate many recommendations from broader literature on sepsis, antimicrobial resistance, and perioperative care. Furthermore, the restriction to English-language publications may have introduced language bias, potentially excluding relevant evidence published in other languages.

Finally, data on newer antimicrobial agents, particularly cefiderocol, in the context of prosthetic joint infection and fracture-related infection remain scarce, being primarily based on case reports and small observational studies. Future prospective multicenter studies should aim to better define patient selection, optimal perioperative prophylaxis, resistance-guided treatment strategies, pharmacokinetics related to bone and biofilm, and clinically relevant outcomes.

## 5. Conclusions

Gram-negative bacteria are generally less common than Gram-positive organisms in most orthopedic infections; however, their clinical significance increases significantly in complex surgical patients. This is particularly true after major trauma, revision surgeries, repeated procedures, prolonged hospital stays, and prior antimicrobial exposure.

The primary challenge lies not only in their prevalence but also in factors such as biofilm formation, host inflammatory activation, and increasingly complicated antimicrobial resistance mechanisms. Infections caused by multidrug-resistant Gram-negative bacteria can severely limit treatment options, highlighting the need for early microbiological diagnosis, effective source control, and personalized antimicrobial therapy.

For anesthesiologists and intensive care physicians, management should extend beyond intraoperative care. It should include identifying high-risk patients, providing appropriate perioperative prophylaxis, early detection of sepsis and organ dysfunction, practicing antimicrobial stewardship, and coordinating multidisciplinary treatment efforts. New agents like cefiderocol are important additions to our treatment options; however, susceptibility testing, resistance mechanisms, and limited clinical evidence of their effectiveness in bone and joint infections should still guide their use in managing orthopedic infections.

## Figures and Tables

**Table 1:** Main microbiological and pathogenic differences between Gram-positive and Gram-negative bacteria relevant to orthopedic infections. **Abbreviations:** CoNS, *coagulase-negative staphylococci*; LPS, lipopolysaccharide; PAMPs, pathogen-associated molecular patterns; TLR4, Toll-like receptor 4; ESBL, extended-spectrum β-lactamase; MDR, multidrug-resistant; PJI, prosthetic joint infection; FRI, fracture-related infection.

Feature	Gram-Positive	Gram-Negative
**Cell envelope**	Thick peptidoglycan	Outer membrane + thin peptidoglycan
**LPS**	Absent	Present
**Implant adhesion**	Particularly relevant in staphylococci	Present, pathogen-dependent
**Biofilm**	Highly relevant	Highly relevant
**Innate immune activation Pathogen-Associated Molecular Patterns (PAMPs) mediated**		Strong LPS/TLR4 component
**Main resistance mechanisms**	e.g., altered targets, β-lactamases	β-lactamases, porin loss, efflux pumps, target modifications
**Typical orthopedic pathogens**	S. aureus, CoNS	*Enterobacterales*, *P. aeruginosa*, *A. baumannii*
**Major clinical concern**	Persistence/chronic implant infection	Persistence + resistance + potential systemic inflammatory response

**Table 2:** Epidemiological characteristics of prosthetic joint infection and fracture-related infection. Abbreviations: CoNS, *coagulase-negative staphylococci*; PJI, prosthetic joint infection.

Clinical Setting	Approximate Incidence	Main Predisposing Factors	Microbiological Considerations
**Primary total hip or knee arthroplasty**	~1–2%	Advanced age, obesity, diabetes, immunosuppression, previous surgery	Gram-positive organisms predominate; Gram-negative pathogens represent a relevant minority
**Revision** **arthroplasty**	Higher than primary arthroplasty	Previous PJI, repeated surgery, prolonged healthcare exposure	Higher probability of polymicrobial and resistant infections
**Closed fracture treated with internal fixation**	~1–2%	Soft-tissue damage, smoking, diabetes, surgical complexity	*S. aureus* and CoNS remain predominant
**Open fracture**	Up to ~30%	Direct contamination, extensive soft-tissue injury, vascular compromise	Broader microbial spectrum, including Gram-negative organisms
**Severe open fracture/Gustilo–Anderson type III**	Up to >30% in selected series	High-energy trauma, tissue devitalization, repeated procedures, prolonged hospitalization	Increased risk of polymicrobial, Gram-negative, and healthcare-associated infections

**Table 3:** International guidelines and consensus recommendations.

Organization/Document	Key Recommendations	Clinical Relevance
EBJIS (2021)—PJI definition [14,29,51,52]	Three diagnostic categories: infection unlikely, likely, or confirmed, integrating clinical, synovial, microbiological and histological findings.	Early identification and diagnostic classification of suspected PJI.
IDSA (2013)—APJI guidelines [14,29,51,52]	Appropriate surgical management, microbiological diagnosis and pathogen-directed antimicrobial treatment.	Coordination of surgical source control and antimicrobial therapy.
IDSA (2024)—AMR guidance [14,29,51,52]	Treatment of resistant Gram-negative infections based on pathogen identification, susceptibility testing and resistance mechanisms.	Selection of appropriate antimicrobial agents in MDR infections.
ESCMID/EUCIC (2023)—Perioperative prophylaxis [14,29,51,52]	Targeted prophylaxis in selected MDR Gram-negative carriers undergoing specific procedures; insufficient evidence for routine extension to orthopedic surgery.	Individualized prophylaxis and antimicrobial stewardship.
International FRI Consensus (2019–2020) [14,29,51,52]	Deep tissue sampling, adequate debridement, implant management and susceptibility-guided antimicrobial treatment.	Surgical source control and microbiological management.

Table 3: Summary of major international guidelines and consensus recommendations relevant to the diagnosis, prevention, and management of Gram-negative orthopedic infections. Abbreviations: EBJIS, European Bone and Joint Infection Society; IDSA, Infectious Diseases Society of America; ESCMID, European Society of Clinical Microbiology and Infectious Diseases; EUCIC, European Committee on Infection Control; PJI, prosthetic joint infection; FRI, fracture-related infection; MDR, multidrug-resistant; AMR, antimicrobial resistance.

## Data Availability

No new data were created or analyzed in this study. Data sharing is not applicable to this article.

## Abbreviations

The following abbreviations are used in this manuscript:

*A. baumannii*	*Acinetobacter baumannii*
CRAB	Carbapenem-resistant *Acinetobacter baumannii*
CoNS	*Coagulase-negative staphylococci*
*E. coli*	*Escherichia coli*
ECDC	European Center for Disease Prevention and Control
ESBL	Extended-spectrum β-lactamase
FRI	Fracture-related infection
GCP	Good Clinical Practice
HAI	Healthcare-associated infection
ICU	Intensive care unit
IMP	Imipenemase
KPC	*Klebsiella pneumoniae* carbapenemase
LPS	Lipopolysaccharide
MDR	Multidrug-resistant
NDM	New Delhi metallo-β-lactamase
OXA-48-like	Oxacillinase-48-like
*P. aeruginosa*	*Pseudomonas aeruginosa*
PAMPs	Pathogen-associated molecular patterns
PJI	Prosthetic joint infection
SANRA	Scale for the Assessment of Narrative Review Articles
SSI	Surgical site infection
TLR2	Toll-like receptor 2
TLR4	Toll-like receptor 4
TNF-α	Tumor necrosis factor alpha
VIM	Verona integron-encoded metallo-β-lactamase
WHO	World Health Organization
XDR	Extensively drug-resistant

## References

[1] Benson E.M., Rutz R.W., Atkins A.C., Carter K.J., Gross E.G., Yeager M., Johnson J.P., Spitler C.A. (2025). Risk Factors and Infection Presentation of Gram-Negative Fracture Related Infections. J. Orthop. Trauma.

[2] Peterlin A.A.N., McNally M., Ferguson J., Gottlieb H., Jensen L.K. (2025). Gram-negative and Gram-positive bacteria induce different osteolysis progression profiles over time in fracture-related infections. Injury.

[3] Sanders P.T.J., Bus M.P.A., Scheper H., van der Wal R.J.P., van de Sande M.A.J., Bramer J.A.M., Schaap G.R., de Boer M.G.J., Dijkstra P.D.S. (2019). Multiflora and Gram-Negative Microorganisms Predominate in Infections Affecting Pelvic Endoprostheses Following Tumor Resection. J. Bone Jt. Surg. Am..

[4] Tsilika M., Ntziora F., Giannitsioti E. (2025). Antimicrobial Treatment Options for Multidrug Resistant Gram-Negative Pathogens in Bone and Joint Infections. Pathogens.

[5] O’Toole R.V., Joshi M., Carlini A.R., Murray C.K., Allen L.E., Huang Y., Scharfstein D.O., O’Hara N.N., Gary J.L., Bosse M.J. (2021). Effect of Intrawound Vancomycin Powder in Operatively Treated High-risk Tibia Fractures: A Randomized Clinical Trial. JAMA Surg..

[6] Russo A., Trecarichi E.M., Torti C. (2022). The role of Gram-negative bacteria in skin and soft tissue infections. Curr. Opin. Infect. Dis..

[7] Agashe V.M., Soman R., Rodrigues C., Shetty A., Sunavala A., Raghuvanshi S., Menon A. (2022). Salvaging lives in carbapenem-resistant Gram-negative osteoarticular and soft tissue infections: Early compromises save lives. Int. Orthop..

[8] Dhillon M.S., Hooda A., Moriarty T.F., Sharma S. (2023). Biofilms-What Should the Orthopedic Surgeon know?. Indian J. Orthop..

[9] Baethge C., Goldbeck-Wood S., Mertens S. (2019). SANRA-a scale for the quality assessment of narrative review articles. Res. Integr. Peer Rev..

[10] Arciola C.R., Campoccia D., Montanaro L. (2018). Implant infections: Adhesion, biofilm formation and immune evasion. Nat. Rev. Microbiol..

[11] Metsemakers W.J., Kuehl R., Moriarty T.F., Richards R.G., Verhofstad M.H.J., Borens O., Kates S., Morgenstern M. (2018). Infection after fracture fixation: Current surgical and microbiological concepts. Injury.

[12] Wang B., Zhang J., Han W., Tang X., Tian F. (2025). Incidence and Risk Factors of Infection After Fracture Fixation: A Multicenter Cohort Study. Orthop. Surg..

[13] Babushkina I.V., Bondarenko A.S., Ulyanov V.Y., Mamonova I.A. (2020). Biofilm Formation by Gram-Negative Bacteria during Implant-Associated Infection. Bull. Exp. Biol. Med..

[14] McNally M., Sousa R., Wouthuyzen-Bakker M., Chen A.F., Soriano A., Vogely H.C., Clauss M., Higuera C.A., Trebše R. (2021). The EBJIS definition of periprosthetic joint infection. Bone Jt. J..

[15] Sousa R., Ribau A., Alfaro P., Burch M.A., Ploegmakers J., McNally M., Clauss M., Wouthuyzen-Bakker M., Soriano A. (2023). The European Bone and Joint Infection Society definition of periprosthetic joint infection is meaningful in clinical practice: A multicentric validation study with comparison with previous definitions. Acta Orthop..

[16] Metsemakers W.J., Kortram K., Morgenstern M., Moriarty T.F., Meex I., Kuehl R., Nijs S., Richards R.G., Raschke M., Borens O. (2018). Definition of infection after fracture fixation: A systematic review of randomized controlled trials to evaluate current practice. Injury.

[17] Morgenstern M., Kühl R., Eckardt H., Acklin Y., Stanic B., Garcia M., Baumhoer D., Metsemakers W.J. (2018). Diagnostic challenges and future perspectives in fracture-related infection. Injury.

[18] Metsemakers W.J., Morgenstern M., McNally M.A., Moriarty T.F., McFadyen I., Scarborough M., Athanasou N.A., Ochsner P.E., Kuehl R., Raschke M. (2018). Fracture-related infection: A consensus on definition from an international expert group. Injury.

[19] Hand T.L., Hand E.O., Welborn A., Zelle B.A. (2020). Gram-Negative Antibiotic Coverage in Gustilo-Anderson Type-III Open Fractures. J. Bone Jt. Surg. Am..

[20] Famada Arboix E., Fischbacher A., Ferrari M.C.M., Borens O., Pérez-Prieto D. (2026). Gram-negative prosthetic joint infections: A retrospective multicentre European study of incidence, risk factors, and treatment outcomes. J. Bone Jt. Infect..

[21] Fantoni M., Borrè S., Rostagno R., Riccio G., Carrega G., Giovannenze F., Taccari F. (2019). Epidemiological and clinical features of prosthetic joint infections caused by gram-negative bacteria. Eur. Rev. Med. Pharmacol. Sci..

[22] Campbell W.R., Li P., Whitman T.J., Blyth D.M., Schnaubelt E.R., Mende K., Tribble D.R. (2017). Multi-Drug-Resistant Gram-Negative Infections in Deployment-Related Trauma Patients. Surg. Infect..

[23] Patel K.H., Gill L.I., Tissingh E.K., Galanis A., Hadjihannas I., Iliadis A.D., Heidari N., Cherian B., Rosmarin C., Vris A. (2023). Microbiological Profile of Fracture Related Infection at a UK Major Trauma Centre. Antibiotics.

[24] Mogensen T.H. (2009). Pathogen recognition and inflammatory signaling in innate immune defenses. Clin. Microbiol. Rev..

[25] Davis K.M., Weiser J.N. (2011). Modifications to the peptidoglycan backbone help bacteria to establish infection. Infect. Immun..

[26] Chanderraj R., Bartek B., Stringer K.A., Tiba M.H., Sjoding M.W., He Y., Nuppnau M., Bongers K.S., Adame M.D., Lou S.S. (2026). Pathogen characteristics are key determinants of distinct host response phenotypes of sepsis. J. Clin. Investig..

[27] Tang A., Shi Y., Dong Q., Wang S., Ge Y., Wang C., Gong Z., Zhang W., Chen W. (2023). Prognostic differences in sepsis caused by gram-negative bacteria and gram-positive bacteria: A systematic review and meta-analysis. Crit. Care.

[28] Righi E., Mutters N.T., Guirao X., Dolores Del Toro M., Eckmann C., Friedrich A.W., Giannella M., Presterl E., Christaki E., Cross E.L.A. (2024). European Society of Clinical Microbiology and Infectious Diseases/European Committee on infection control clinical guidelines on pre-operative decolonization and targeted prophylaxis in patients colonized by multidrug-resistant Gram-positive bacteria before surgery. Clin. Microbiol. Infect..

[29] Righi E., Mutters N.T., Guirao X., Del Toro M.D., Eckmann C., Friedrich A.W., Giannella M., Kluytmans J., Presterl E., Christaki E. (2023). ESCMID/EUCIC clinical practice guidelines on perioperative antibiotic prophylaxis in patients colonized by multidrug-resistant Gram-negative bacteria before surgery. Clin. Microbiol. Infect..

[30] Tamma P.D., Bonomo R.A., Heil E.L., Justo J.A., Satlin M.J., Mathers A.J. (2026). Infectious Diseases Society of America 2026 Guidance on the Treatment of Antimicrobial-Resistant Gram-Negative Infections. Clin. Infect. Dis..

[31] Tamma P.D., Aitken S.L., Bonomo R.A., Mathers A.J., van Duin D., Clancy C.J. (2022). Infectious Diseases Society of America Guidance on the Treatment of AmpC β-Lactamase-Producing Enterobacterales, Carbapenem-Resistant Acinetobacter baumannii, and Stenotrophomonas maltophilia Infections. Clin. Infect. Dis..

[32] Achatz V., Sebastian S., Mitterer J.A., Tatar E.D., Akcicek E., Sadoghi P., Hofstaetter J.G. (2026). Beyond Gram-Negative and Gram-Positive Bacteria: Virulence as a Determinant of Clinical Outcome in Periprosthetic Joint Infections. J. Arthroplast..

[33] Sati H., Carrara E., Savoldi A., Hansen P., Garlasco J., Campagnaro E., Boccia S., Castillo-Polo J.A., Magrini E., Garcia-Vello P. (2025). The WHO Bacterial Priority Pathogens List 2024: A prioritisation study to guide research, development, and public health strategies against antimicrobial resistance. Lancet Infect. Dis..

[34] Papadopoulos A., Ribera A., Mavrogenis A.F., Rodriguez-Pardo D., Bonnet E., Salles M.J., Dolores Del Toro M., Nguyen S., Blanco-García A., Skaliczki G. (2019). Multidrug-resistant and extensively drug-resistant Gram-negative prosthetic joint infections: Role of surgery and impact of colistin administration. Int. J. Antimicrob. Agents.

[35] Eckmann C., Wagner N.M. (2026). Perioperative surgical prophylaxis in the era of multidrug-resistant gram-negatives. Curr. Opin. Infect. Dis..

[36] Giacobbe D.R., Karaiskos I. (2020). Stewardship of Antibiotics for Multidrug-Resistant Gram-Negative Bacteria. Antibiotics.

[37] Dunbar S.A., Benavides M., Gardner C. (2025). Advances in diagnostic methods for detection of bloodstream pathogens and antibiotic resistance determinants. Front. Cell. Infect. Microbiol..

[38] Kannan M., Jobby R., Rajasekharan S.K., Arya R., Ravichandran V. (2025). Molecular Framework of Multidrug Resistance in Pseudomonas aeruginosa. Curr. Microbiol..

[39] Meschiari M., Asquier-Khati A., Tiseo G., Luque-Paz D., Murri R., Boutoille D., Falcone M., Mussini C., Tattevin P. (2024). Treatment of infections caused by multidrug-resistant Gram-negative bacilli: A practical approach by the Italian (SIMIT) and French (SPILF) Societies of Infectious Diseases. Int. J. Antimicrob. Agents.

[40] Medioli F., Bacca E., Faltoni M., Burastero G.J., Volpi S., Menozzi M., Orlando G., Bedini A., Franceschini E., Mussini C. (2022). Is It Possible to Eradicate Carbapenem-Resistant Acinetobacter baumannii (CRAB) from Endemic Hospitals?. Antibiotics.

[41] De Backer D., Deutschman C.S., Hellman J., Myatra S.N., Ostermann M., Prescott H.C., Talmor D., Antonelli M., Pontes Azevedo L.C., Bauer S.R. (2024). Surviving Sepsis Campaign Research Priorities 2023. Crit. Care Med..

[42] Oczkowski S., Alshamsi F., Belley-Cote E., Centofanti J.E., Hylander Møller M., Nunnaly M.E., Alhazzani W. (2022). Surviving Sepsis Campaign Guidelines 2021: Highlights for the practicing clinician. Pol. Arch. Intern. Med..

[43] Patel K.M., Mears S.C., Barnes C.L., Stambough J.B., Stronach B.M. (2022). Sepsis and Total Joint Arthroplasty. Orthop. Clin. N. Am..

[44] Liu H., Xu C., Hu Q., Wang Y. (2025). Sepsis-induced cardiomyopathy: Understanding pathophysiology and clinical implications. Arch. Toxicol..

[45] Tippu A.R., Mazari M.I., Ahmad M.S., Faisal M.U., Mughal N.S., Bhatti R.A. (2024). Prevalence of post-operative wound infections among orthopaedics patients with surgical implant tertiary care hospital. J. Ayub Med. Coll. Abbottabad.

[46] Gatti M., Viaggi B., Rossolini G.M., Pea F., Viale P. (2021). An Evidence-Based Multidisciplinary Approach Focused on Creating Algorithms for Targeted Therapy of Infection-Related Ventilator-Associated Complications (IVACs) Caused by Pseudomonas aeruginosa and Acinetobacter baumannii in Critically Ill Adult Patients. Antibiotics.

[47] Gatti M., Viaggi B., Rossolini G.M., Pea F., Viale P. (2021). An Evidence-Based Multidisciplinary Approach Focused at Creating Algorithms for Targeted Therapy of BSIs, cUTIs, and cIAIs Caused by Enterobacterales in Critically Ill Adult Patients. Infect. Drug Resist..

[48] Chambers M.M., Gutowski C.J., Doktor K. (2023). Cefiderocol for Stenotrophomonas maltophilia prosthetic joint infection: A case report. Ther. Adv. Infect. Dis..

[49] Alamarat Z.I., Babic J., Tran T.T., Wootton S.H., Dinh A.Q., Miller W.R., Hanson B., Wanger A., Gary J.L., Arias C.A. (2020). Long-Term Compassionate Use of Cefiderocol To Treat Chronic Osteomyelitis Caused by Extensively Drug-Resistant Pseudomonas aeruginosa and Extended-Spectrum-β-Lactamase-Producing Klebsiella pneumoniae in a Pediatric Patient. Antimicrob. Agents Chemother..

[50] Zingg S., Nicoletti G.J., Kuster S., Junker M., Widmer A., Egli A., Hinic V., Sendi P., Battegay M., Bättig V. (2020). Cefiderocol for Extensively Drug-Resistant Gram-Negative Bacterial Infections: Real-world Experience From a Case Series and Review of the Literature. Open Forum Infect. Dis..

[51] Tamma P.D., Heil E.L., Justo J.A., Mathers A.J., Satlin M.J., Bonomo R.A. (2024). Infectious Diseases Society of America 2024 Guidance on the Treatment of Antimicrobial-Resistant Gram-Negative Infections. Clin. Infect. Dis..

[52] Depypere M., Kuehl R., Metsemakers W.J., Senneville E., McNally M.A., Obremskey W.T., Zimmerli W., Atkins B.L., Trampuz A. (2020). Recommendations for Systemic Antimicrobial Therapy in Fracture-Related Infection: A Consensus From an International Expert Group. J. Orthop. Trauma.

[53] Konda S.R., Dedhia N., Ganta A., Behery O., Haglin J.M., Egol K.A. (2022). Risk Factors for Gram-Negative Fracture-Related Infection. Orthopedics.

[54] Burastero G.J., Orlando G., Santoro A., Menozzi M., Franceschini E., Bedini A., Cervo A., Faltoni M., Bacca E., Biagioni E. (2022). Ceftazidime/Avibactam in Ventilator-Associated Pneumonia Due to Difficult-to-Treat Non-Fermenter Gram-Negative Bacteria in COVID-19 Patients: A Case Series and Review of the Literature. Antibiotics.

[55] Kalbian I.L., Goswami K., Tan T.L., John N., Foltz C., Parvizi J., Arnold W.V. (2020). Treatment Outcomes and Attrition in Gram-Negative Periprosthetic Joint Infection. J. Arthroplast..

[56] Hiddema J.S. (2023). Periprosthetic joint infection:A South African perspective. S. Afr. Med. J..

[57] Honkanen M. (2025). Risk of orthopaedic implant infection during bacteraemia. Apmis.

[58] Bonnet E., Lourtet-Hascoët J. (2025). Combination Antibiotic Therapy for Orthopedic Infections. Antibiotics.

[59] Wang L., Zhu J., Chen L., Du H. (2024). Cefiderocol: Clinical application and emergence of resistance. Drug Resist. Updates.

[60] Shi T.B., Fang X.Y., Wang C.X., Cai Y.Q., Li W.B., Zhang W.M. (2020). Rare Occurrence of Acute Hematogenous Periprosthetic Joint Infection Due to Fusobacterium Nucleatum in the Background of a Dental Procedure: A Case Report. Orthop. Surg..

[61] Papadakis S.A., Lykos S., Pallis D., Mantzanas P., Paraskevopoulos N., Kamariotis S. (2026). Periprosthetic Hip Joint Infection Involving Fusobacterium nucleatum With Subsequent Isolation of Acinetobacter baumannii: A Case Report. Cureus.

[62] Rempenault C., Pagis V., Noussair L., Berbescu S., Duran C., Bouchand F., de Laroche M., Salomon E., Nich C., Bauer T. (2021). Treatment of bone and joint infections by ceftazidime/avibactam and ceftolozane/tazobactam: A cohort study. J. Glob. Antimicrob. Resist..

[63] Diarra A., Degrendel M., Eberl I., Ferry T., Jaffal K., Escaut L., Khati A.A., Taar N., Courjon J., Deconinck L. (2025). Real-Life Cefiderocol Use in Bone and Joint Infection: A French National Cohort. Antibiotics.

